# Effect of Different Hydration Time on Carbonation Degree and Strength of Steel Slag Specimens Containing Zeolite

**DOI:** 10.3390/ma13173898

**Published:** 2020-09-03

**Authors:** Xiong Zhang, Jun Chang

**Affiliations:** School of Civil Engineering, Dalian University of Technology, Dalian 116024, China; zhangxiong520@mail.dlut.edu.cn

**Keywords:** steel slag, zeolite, pozzolanic reaction, carbonation degree, CaCO_3_, C-S-H

## Abstract

Steel slag partially substituted by zeolite (SZ) was beneficial for improving the compressive strength and carbonation degree of SZ specimens after a combined curing (hydration and then carbonation) process due to pozzolanic reaction between them. By previous work results, the zeolitic substitution ratios of 5 wt.% and 15 wt.% in steel slag specimens (SZ5 and SZ15) gained the optimum compressive strength and carbonation degree, respectively, after 1 day hydration and then 2 h carbonation. This study investigated the effect of previous hydration time (1, 3, 7, 14, and 196 days) on carbonation degree and strength of SZ specimens after subsequent carbonation curing. Two zeolitic substitution ratios (5 wt.% and 15 wt.%) were selected and pure steel slag specimens were also prepared as controls. Compressive strength results revealed that the optimum hydration curing time was 1 day and the optimum zeolitic substitution ratio was 5 wt.%. The pozzolanic reaction happened in SZ specimens was divided into early and late pozzolanic reaction. In the late hydration, a new mineral, monocarboaluminate (AFmc) was produced in SZ15 specimens, modifying the carbonation degree and strength further. And the mechanism of pozzolanic reaction in early and late hydration in SZ specimens was explained by several microscopic test methods.

## 1. Introduction

CO_2_ is one of the main causes of global warming. For reducing the amount of CO_2_ emission, many renewable energy sources, e.g., wind and solar energy, are used to replace conventional fossil fuels. Meanwhile, carbon capture and storage technology has been applied for CO_2_ disposal in the atmosphere already [1,2]. And one feasible method for long-term storage of CO_2_ is mineral carbonation, i.e., the process of silicate Ca/Mg minerals reacting with CO_3_^2−^ to convert into stable carbonates [3]. Due to the lack of natural feedstocks, the alkaline industrial solid waste, such as steel slag [4], would be considered as economical and ecological alternative resource for mineral consumption.

Steel slag is a by-product obtained by the process of steelmaking of iron ores with limestone [5]. On average, the output amount of steel slag is around 200 kg for manufacturing per tonne of steel [6]. According to the statistics from Euroslag. the production amount of steel slag reached 15.7 Mt and the total use amount reached 11.5 Mt in Europe 2018 (utilization ratio, 73%) [7]. However, the utilization ratio of steel slag in China was only 10–20% by the statistics from the industrial solid waste network in 2017. The low utilization ratio of steel slag was due to the wide variability of composition, the poor hydraulic property [8,9], the inordinate volume expansion [10,11], and the high trace metal ions leaching [12]. However, steel slag performed well after activated by the carbonation process [13]. Furthermore, basic oxygen furnace slag (BOFS), a type of steel slag, has a higher carbonation activity than that of ladle furnace slag (LFS) and electric arc furnace slag (EAFS) [14,15].

Accelerated carbonation is an innovative method, in which Ca/Mg-silicates minerals react with CO_2_ and moisture to form calcium or magnesium carbonate in Equation (1) [4]. It can increase the compressive strength of steel slag products dramatically in a few hours by forming calcium carbonate. And Shao et al. [16] proposed the combined-curing process (carbonation 2 h and then hydration 28 days) to enhance the compressive strength of steel slag compacts further. To improve the carbonation degree, many researchers investigated the influence of carbonations by controlling parameters such as CO_2_ concentration, partial pressure, humidity, compacting pressure, slag fineness, carbonation duration, and temperature. Except for these methods, steel slag partially substituted by zeolite was also beneficial to modify the compressive strength and carbonation degree by the previous study.

(Ca, Mg)SiO_3_ + CO_2_ → (Ca, Mg)CO_3_ + SiO_2_ (1)

Natural zeolites containing alumina silicate are used as supplementary cementitious materials in cement due to their pozzolanic activity. Canpolat et al. [17] investigated that after 2 and 7 days hydration, the optimum zeolitic substitution ratio in Portland cement for compressive strength was 5%. However, prolonging the hydration time to 28 days and 90 days, the optimum substitution ratio became 20%. Yılmaz et al. [18] also found a similar phenomenon. And Samimi et al. [19] found that the self-compacting concrete based on the zeolite mixture was more vulnerable to carbonation. Steel slag has similar minerals (β-C_2_S, C_4_AF, and Ca(OH)_2_) compared with Portland cement. To improve the carbonation degree of steel slag, a novel and economical method, zeolite partial replaced steel slag, was proposed. Previous study results had already found that after 1 day hydration and 2 h carbonation, the optimum substitution ratio of steel slag by zeolite for compressive strength and carbonation degree was 5% and 15%, respectively. However, the optimum hydration time before carbonation of slag partially substituted by zeolite (SZ) specimens has not been investigated systematically. With the extension of curing time, the pozzolanic reaction extent would increase, increasing the amount of hydration product, such as C–S–H gel [20,21], to influence the carbonation degree and compressive strength of SZ specimens. Meanwhile, whether the optimum zeolitic substitution proportion would transfer with prolonging the hydration time was also worthy to investigate.

This paper investigated the effect of previous hydration time (1, 3, 7, 14, 196 days) on carbonation degree and strength of SZ specimens after subsequent carbonation curing. Two zeolitic substitution ratios (5 and 15 wt.%) were chosen by previous work results. At the same time, pure steel slag specimens were prepared as control. The compressive strength value and carbonation degree of SZ specimens after hydration (HSZ) or combined curing (hydration and then 2 h carbonation, CSZ) processes were tested. Pozzolanic reaction before carbonation in SZ specimens was beneficial for enhancing the compressive strength and carbonation degree of SZ specimens by previous work. The ultimate goal of the devised processing route in this study was to find the optimum hydration time before carbonation of SZ specimens and then explained the combined-curing reaction mechanism. The optimum curing process was 1 day hydration and then 2 h carbonation, considering the carbonation degree and strength of SZ specimens both. Pozzolanic reaction in SZ specimens has happened. It was divided into early and late pozzolanic reaction. In the late hydration, a new mineral, monocarboaluminate (AFmc), was detected in SZ15 specimens and then participated in the carbonation reaction to modify the carbonation degree and strength of SZ specimens further. And the mechanism of pozzolanic reaction in early and late hydration in SZ specimens was proved further by several microscopic test methods.

## 2. Materials and Methods

### 2.1. Materials

**Figure 1:**
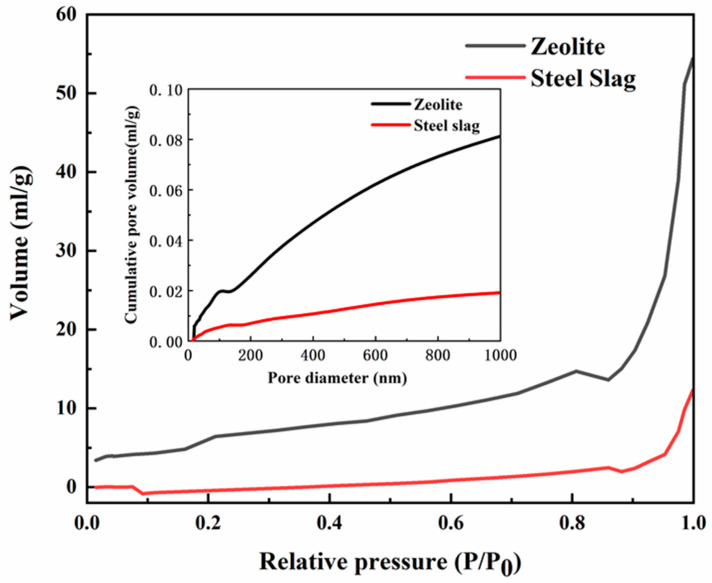
Nitrogen adsorption–desorption isotherms on steel slag and zeolite.

**Figure 2:**
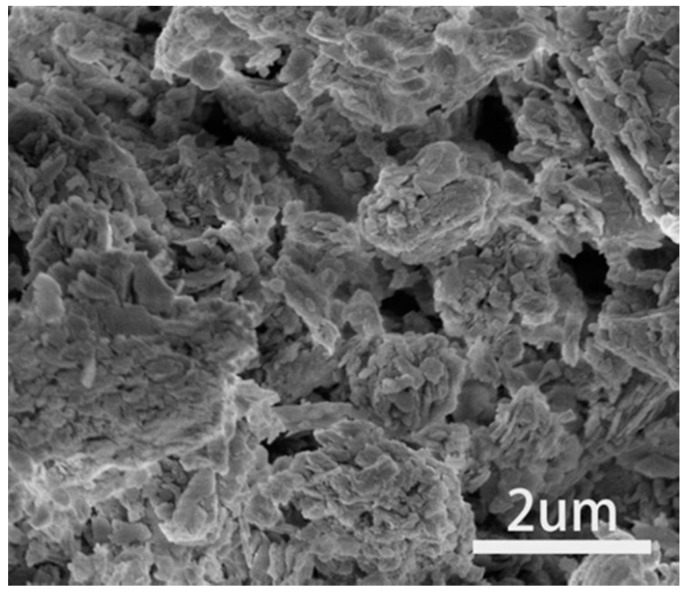
Scanning electron microscopy (SEM) image of natural zeolite.

The steel slag and zeolite used in this work are respectively supplied by Benxi Steel Group Corporation and Tubaoying Zeolite Group Corporation in China. Raw materials were ground in a ball mill and then passed through an 80 μm sieve for improving the carbonation curing efficiency by changing the particle size [22]. The particle size of steel slag powder was measured by a laser diffraction particle size analyzer (NKT6200, Liaoning, China) and the values of D10 (i.e., the diameter of the particles with 10% passing, similarly hereinafter), D50 and D90 were 1.69, 7.92, and 77.02 μm, respectively. For zeolite powder, the values of D10, D50, and D90 were 2.39, 31.16, and 84.61 μm. The pore size diagram of raw materials was obtained by nitrogen adsorption–desorption isotherms and pore volume curve of Brunner Emmett Teller (BET-AUTO SORB-1-MP, Boynton Beach, FL, USA) as shown in Figure 1. The results showed that the microporous volume of zeolite was 4.3 times that of steel slag. The microstructure of zeolite was obtained by Scanning Electron Microscopy (SEM, FEI NOVA NanoSEM 450, Chelmsford, MA, USA) as shown in Figure 2. The chemical compositions of raw materials were determined by X-ray fluorescence (XRF-1800, Shimadzu, Japan) spectrometry and the results were present in Table 1. Phase identification and quantification in raw materials were determined by the X-ray diffraction (XRD) technique with Diffract EVA software (Bruker, Karlsruhe, Germany) and TOPAS4.2 software (Bruker, Karlsruhe, Germany) respectively, and the results were shown in Table 2.

### 2.2. Samples Preparation

**Figure 3:**
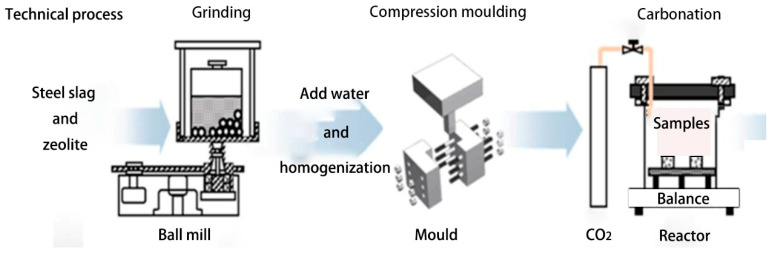
Schematic illustration used to produce steel slag cubes containing different ratios of zeolite.

To investigate the effect of previous hydration time on carbonation degree and strength of SZ specimens after subsequent carbonation curing, five hydration curing periods (1, 3, 7, 14, and 196 days) were designed. Zeolitic substitution ratios of 0, 5, and 15 wt.% were selected by the previous study. The mixture compositions of the test compacts in detail were present in Table 3. As shown in Figure 3, weighed materials with designing zeolitic substitution ratios were dry-mixed for 2 min, then 13 wt.% deionized water (i.e., water-to-solid ratio of 0.13) was added. After 5 min of stirring, the fresh paste was put into the stainless-steel mold for pressing into the test cubes (20 mm × 20 mm × 20 mm) until reaching 8 MPa molding pressure. And then the prepared test cubes were put into the standard curing chamber for 1, 3, 7, 14, and 196 days hydration curing, named as HSZ0-1d, HSZ5-1d, and HSZ15-1d to HSZ0-196d, HSZ5-196d, and HSZ15-196d, respectively. Then they were put into the carbonation reactor with a CO_2_ concentration of 99% and a constant pressure of 0.2 MPa for 2 h carbonation curing, named as CSZ0-1d, CSZ5-1d, and CSZ15-1d to CSZ0-196d, CSZ5-196d, and CSZ15-196d, respectively, and then put them into the vacuum drying oven to constant weight at 50 °C.

### 2.3. Strength Gained and Carbonation Degree

The compressive strength of HSZ and CSZ compacts were tested by a 50 kN compression testing machine with a loading rate of 0.5 mm/min. For each group, at least three compacts were tested and then averaged.

The technique used to assess the carbonation degree of the SZ specimens in this study was the thermo-gravimetry analysis (TGA) method. This method was carried out by Mettler Toledo TGA/DSC1 with N_2_ flow (rate, 50 mL/min). The dry specimens powders weighed 20 ± 1 mg were put in corundum crucibles and then heated from 50 to 1000 °C with a constant heating rate of 10 °C/min. The mass loss of hydration and carbonation products was determined by the differential thermogravimetry (DTG) curve by STARe software (Mettler toledo, Zurich, Switzerland). The mass loss after 500 °C was determined by the decomposition of calcium carbonate in the steel slag matrix [23]. When the CaO content and weight loss percentage of CO_2_ were determined, the carbonation degree of steel slag could be obtained by Equation (2) [10,24].

(2) $$\zeta \mathrm{CaO}=\frac{\frac{{\mathrm{CO}}_{2}\left(\mathrm{wt}\%\right)}{100-{\mathrm{CO}}_{2}\left(\mathrm{wt}\%\right)}\times \frac{1}{{\mathrm{MW}}_{{\mathrm{CO}}_{2}}\left(g/\mathrm{mol}\right)}}{{\mathrm{CaO}}_{\mathrm{total}}{(\mathrm{wt}\%)/\mathrm{MW}}_{\mathrm{CaO}}\left(g/\mathrm{mol}\right)}\times 100$$

where MWCO_2_ and MWCaO are the molar weight of CO_2_ (44 g/mol) and CaO (56 g/mol); CaO_total_ is the weight fraction of the total CaO content (47.21 wt.%) which performed by XRF (see Table 1). Meanwhile, zeolite does not have carbonation activity, so the calculation formula of carbonation degree of SZ specimens was presented in Equation (3).

(3) $$\zeta SS=\frac{\zeta \mathrm{CaO}}{1-\mathrm{WZ}}$$

where *ζSS* is the carbonation degree of the steel slag specimens deducting the zeolite content, and WZ is the zeolitic substitution proportion. ζCaO is the carbonation degree of steel slag obtained from Equation (2).

### 2.4. Pozzolanic Reaction

To investigate the extent change of pozzolanic reaction in SZ specimens after prolonging the hydration curing time before carbonation, the Fourier transform infrared spectroscopy (FT-IR) was obtained on a Bruker EQUINOX55 (Karlsruhe, Germany) at room temperature. Samples were prepared by mixing 1 mg of sample in 300 mg of KBr. The spectral analysis was performed over the range of 4000–400 cm^−1^ at a resolution of 4 cm^−1^. The infrared spectrum was recorded and stored by spectroscopic software (OPUS, version 5.5). The amount of C–S–H gel could be observed roughly according to the stretching vibration strength of different groups to judge whether the pozzolanic reaction occurred or not. The mass loss at the range of 100–300 °C in TG/DTG curves could also quantify the pozzolanic reaction extent after different hydration curing time.

### 2.5. XRD Analysis

After hydration or combined-curing process, the mineral phases in SZ specimens were identified and quantified by the X-ray diffraction (XRD) technique. The diffraction data was determined by Bruker D8 Advance X-ray Powder Diffractometer (Karlsruhe, Germany) with Cu Kα radiation (*λ* = 0.154 nm, 40 kV and 40 mA, scan range 5–80° 2*θ*, 0.02°, and 0.5 s). Phase identification and quantification were determined by Diffract EVA software and TOPAS4.2 software. Meanwhile, the Rietveld refinement quantitative analysis (QXRD, quantitative X-ray diffraction, Bruker, Karlsruhe, Germany) [25] was performed. 10 wt.% ZnO was mixed to determine the amorphous and crystalline non-quantified (*ACn*) content [25]. The calculation formula was presented in Equation (4) [25]:

(4) $$ACn=\frac{1-W_{st}/R_{st}}{100-W_{st}}\times 10^{4}\%$$

where *W_st_* is the weight fraction of added ZnO (10 wt.%) and *R_st_* is the Rietveld refined weight fraction of ZnO. Meanwhile, the crystal structure files (crystallography open database (COD) codes, see Table 4) of the different phases were used to simulate the diffraction datum. The *R_wp_* values of the profile refinement in the Rietveld refinement process were obtained to evaluate the quality of the fits. Generally, the results are considered to be reliable while *R_wp_* value < 15% [26,27].

### 2.6. Microstructural Analysis

To study the effects of different hydration curing periods on the pore size distribution of CSZ specimens, a mercury intrusion porosimeter (MIP, AUTOPORE IV 9500 series, Micromeritics Instrument Corp, Downers Grove, IL, USA) method was performed. The tested specimens were broken into 3–5 mm pieces and the total weight was approximately 1.5 g. The pieces were dried in a vacuum chamber at 50 °C for 24 h to remove absorbed water in pores.

The microstructure of HSZ and CSZ specimens with different hydration curing time was determined by FEI NOVA Nano-SEM 450 field emission scanning electron microscopy (FE-SEM, FEI company, Chelmsford, MA, USA).

## 3. Results and Discussions

### 3.1. Hydration and Carbonation Behavior of HSZ and CSZ Specimens

**Figure 4:**
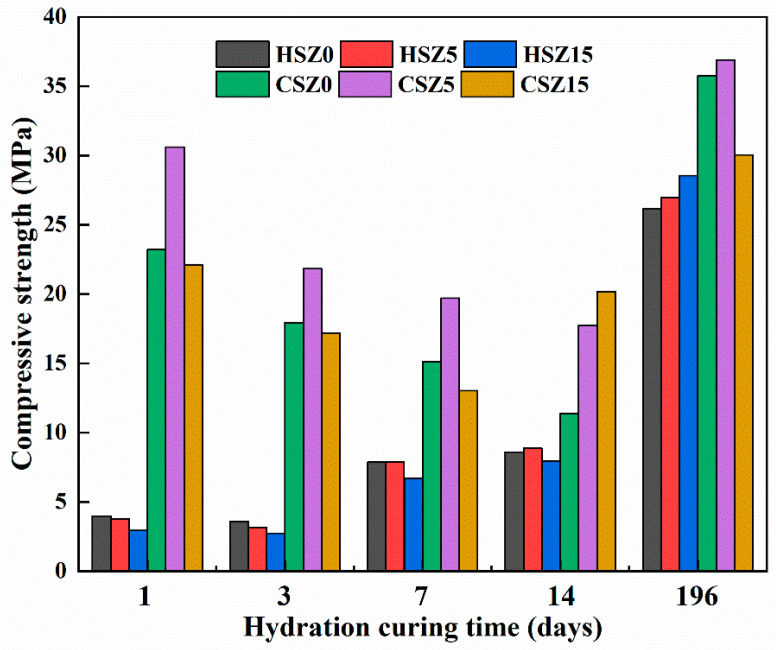
Compressive strength of SZ specimens after “H” or “C”. (H: hydration at different times; C: hydration at different times and then 2 h carbonation).

The compressive strength values of HSZ and CSZ specimens were shown in Figure 4. Steel slag with zeolite (SZ) specimens had shown a latent hydraulic behavior. In the first 3 days, the strength of SZ specimens gained slightly. Meanwhile, the sequence of compressive strength values was HSZ0 > HSZ5 > HSZ15. When the hydration time increased to 7 days, all strength value nearly doubled it. And the compressive strength of HSZ5 was equal to that of HSZ0. After 14 days hydration, the strength value of HSZ5 was slightly higher than that of HSZ0. The increment of strength value was beneficial from zeolite with pozzolanic activity. After 196 days hydration, the sequence of compressive values was HSZ15 > HSZ5 > HSZ0. It illustrated that a higher zeolite ratio participated in a pozzolanic reaction to modify the strength in HSZ15 by prolonging the hydration curing time. Obviously, in the first 7 days hydration, steel slag substituted by zeolite partially got the strength loss compared with HSZ0 due to its high porosity. However, after prolonging hydration time to 14 and 196 days, the strength of SZ specimens started to increase due to the pozzolanic reaction of zeolite in an alkaline environment created by steel slag. Meanwhile, the strength value of HSZ5-14d performed higher than HSZ15-14d. However, the sequence of strength value was opposite for HSZ5-196d and HSZ15-196d. It illustrated that excess zeolite participated in the pozzolanic reaction with the prolonging hydration curing time.

Figure 4 also showed the strength of SZ specimens after carbonation. Obviously, from these values, the combined-curing process helped improve the compressive strength of SZ specimens further. However, the strength contribution ratio from carbonation was decreased with the increment of hydration time. Compared with other hydration curing time, the growth ratio of compressive strength in CSZ-1d specimens was highest. Meanwhile, CSZ5-1d performed higher compressive strength than that of CSZ0-1d. Previous work had proved that a 5% substitution ratio of steel slag by zeolite was the optimum ratio for the compressive strength of steel slag matrix after 1d hydration and 2 h carbonation. The regular sequence of carbonation strength value about CSZ-1d, CSZ-3d, and CSZ-7d specimens was consistent with previous work (CSZ5 > CSZ0 > CSZ15). However, the strength of CSZ15-14d was higher than that of CSZ5-14d. It might indicate that unreacted crystalline zeolite continued to react with Ca-bearing minerals in steel slag to modify the carbonation degree and pore structure. With the hydration time extended to 196 days, the strength of specimen was major provided by hydration and pozzolanic reaction even after carbonation curing, owing to the excess consumption of β-C_2_S and Ca(OH)_2_. Considering the factors of compressive strength and carbonation degree comprehensively, the optimum hydration time before carbonation and zeolitic substitution ratio in SZ specimens were 1 day and 5%, respectively.

### 3.2. Phase Analysis

**Figure 5:**
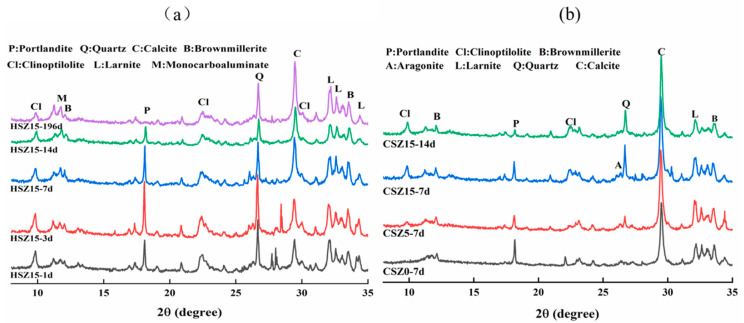
X-ray diffraction (XRD) curves of SZ specimens after the “H” or “C” process. (**a**) evolution of minerals peak intensity in SZ15 after the “H” process; (**b**) evolution of minerals peak intensity in SZ specimens after the “C” process. (H: hydration at different times; C: hydration at different time and subsequent 2 h carbonation).

X-ray diffraction curves of differently processed SZ specimens are shown in Figure 5a summarized the XRD curves of HSZ15 after different hydration curing times. After prolonging the hydration curing time, the relative intensities of the clinoptilolite and larnite (β-C_2_S) peaks decreased slightly, owing to the hydration reaction and pozzolanic reaction in Equations (5)–(8) [28,29,30,31]. The intensity of portlandite (Ca(OH)_2_) peak enhanced in the first 7 days and subsequently weakened, signifying the consumption speed of crystalline clinoptilolite during the pozzolanic reaction in steel slag matrix was lower than that of β-C_2_S hydration in the first 7 days. However, prolonging the hydration time to 14 days, the intensities of clinoptilolite and portlandite peaks weakened apparently. It indicated that pozzolanic reaction played a major role in HSZ specimens in this hydration period, because the partially broken skeleton structure of the zeolite caused it easier to react in an alkaline environment to increase the consumption of Ca(OH)_2_ and clinoptilolite. After 196 days hydration, the peak of portlandite disappeared totally in HSZ specimens. And the intensity of a new peak at 2θ = 11.8° enhanced gradually with lengthening hydration curing time, owing to the hydration reaction in Equation (9) [30]. The new mineral monocarboaluminate (AFmc) was produced, and the Al(OH)_3_ was provided by zeolite in pozzolanic reaction in Equation (8). Figure 5b presented the XRD curves of CSZ specimens after different hydration curing time and then 2h carbonation. Carbonation was generally found to cause a more visible reduction in peak intensities of portlandite and larnite. Also, the peak of newly generated mineral, monocarboaluminate, disappeared after carbonation curing in CSZ specimens. On the contrary, the intensity of CaCO_3_ peak enhanced dramatically. The predominant CaCO_3_ intense peak was related to calcite and the trace of aragonite was also detected.

2CaO·SiO_2_ + mH_2_O → xCaO·SiO_2_·yH_2_O + (2−x)Ca(OH)_2_ (5)

SiO_2_ (active) + Ca(OH)_2_ (aq) → CaO·SiO_2_ (aq) (6)

^3+^Si−O−Si^3+^ + 6OH^−^ → 2[SiO(OH)_3_] ^−^ (7)

^3+^Si−O−Al^3+^ + 7OH^−^ → [SiO(OH)_3_]^−^ + [Al(OH)_4_]^−^ (8)

2[SiO(OH)_3_]^−^ + Ca^2+^ → xCaO·SiO_2_·yH_2_O (9)

3Ca(OH)_2_ + CaCO_3_ + 2Al(OH)_3_ + 5H_2_O → 4CaO·Al_2_O_3_·CO_2_·11H_2_O (10)

**Figure 6:**
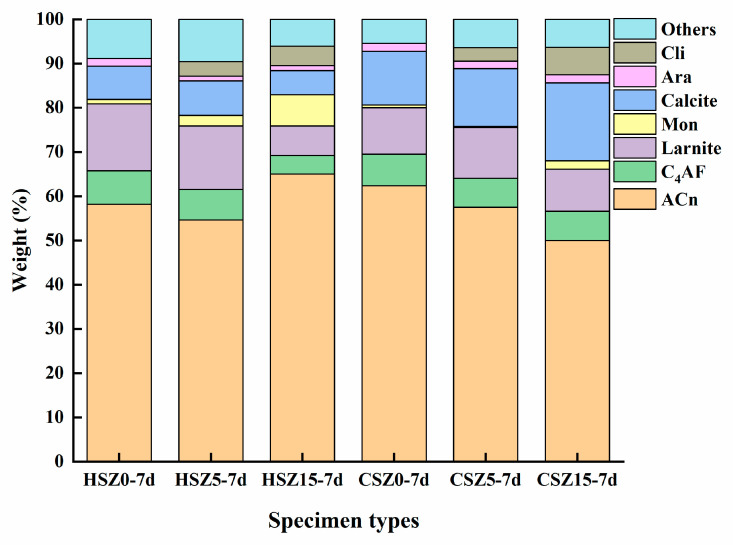
Evolution of minerals SZ specimens during the “H” and “C” process. (H: 7 days hydration; C: 7 days hydration and then 2 h carbonation).

For better understanding the strength development mechanism of SZ samples after hydration and carbonation, The Rietveld method (QXRD) was carried out on the HSZ-7d and CSZ-7d specimens. The results were shown in Figure 6. After 7 days hydration, monocarboaluminate produced in HSZ specimens, especially in HSZ15-7d due to the higher content of zeolite, providing sufficient Al ion for reaction in Equation (10). After 2 h carbonation, the CaCO_3_ content in CSZ5-7d and CSZ15-7d was 12.1% and 45.3% higher than that of CSZ0-7d, respectively. Meanwhile, monocarboaluminate nearly disappeared in CSZ15-7d, owing to the reaction in Equation (11) [32,33]. Compared with HSZ-7d, the reduction of ACn amount in CSZ15-7d was owing to the C–S–H carbonation in Equation (12). Combined the strength value of CSZ specimens and XRD analysis, it revealed that 5 wt.% substitution of steel slag by zeolite performed optimum strength in CSZ-1d, CSZ-3d, and CSZ-7d. However, the optimum substitution ratio changed from 5 to 15 wt.% in CSZ-14d. Combined with XRD analysis, the weaken intensities of clinoptilolite peaks in HSZ-14d indicated that a higher amount of zeolite participated in the pozzolanic reaction in Equations (6)–(10). The increasing amount of C–S–H gel and monocarboaluminate was beneficial for improving the compressive strength and carbonation degree of CSZ specimens.

4CaO·Al2O_3_·CO_2_ 11H_2_O + 3CO_2_ → 4CaCO_3_ +2Al(OH)_3_ + 8H_2_O (11)

C_x_–S–H_y_ + xCO_2_ → SiO_2_ ·yH_2_O + xCaCO_3_ (12)

### 3.3. Thermal and Carbonation Degree Analysis

**Figure 7:**
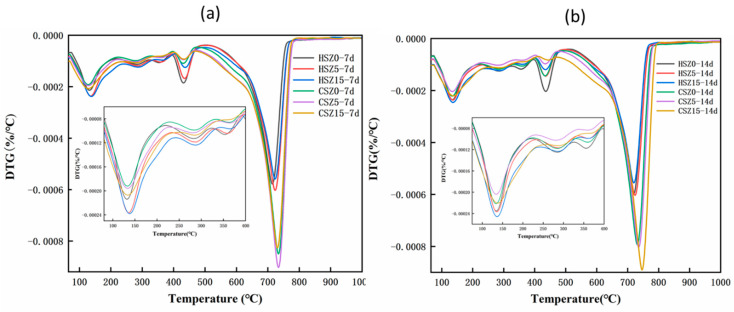
Differential thermogravimetry (DTG) curves of SZ specimens after “H” or “C”. (H: hydration at different times; C: hydration at different times and subsequent 2 h carbonation). (**a**) DTG curves of HSZ-7d and CSZ-7d; (**b**) DTG curves of HSZ-14d and CSZ-14d.

Figure 7 displayed the DTG curves of SZ specimens after “H” or “C”. Based on DTG curves and other researches, the evident mass-loss temperature ranges in 50–345 °C, 345–410 °C, 410–500 °C, and 500–850 °C were identified as the decomposition of C–S–H, Mg(OH)_2_, Ca(OH)_2_, and CaCO_3_ [16,28], respectively. Meanwhile, the decomposition temperature range of AFmc also concluded in 150–250 °C [34]. After 7 days hydration, the reduction amounts of Ca(OH)_2_ in HSZ5 and HSZ15 were 7.0 and 6.8 wt.% higher than that of HSZ0 (considering the Ca(OH)_2_ loss amount in zeolite substitution process). It revealed the partial amount CH participated in pozzolanic reaction. Compared to HSZ0, higher amount of C–S–H product generated in HSZ5 and HSZ15 specimens. Meanwhile, in the range of 150–250 °C, the amount of AFmc increased in HSZ15-7d specimens and then decreased after 2 h carbonation, owing to the reaction in Equations (10) and (11). This phenomenon was consistent with QXRD results. And also, after 2 h carbonation, the CaCO_3_ amount enhanced dramatically and the highest CaCO_3_ amounts in Figure 7a,b were CSZ5-7d and CSZ15-14d, respectively. Combined with the strength results (see Figure 4) and previous research, the increment of CaCO_3_ is beneficial for the strength development of carbonation SZ specimens.

**Figure 8:**
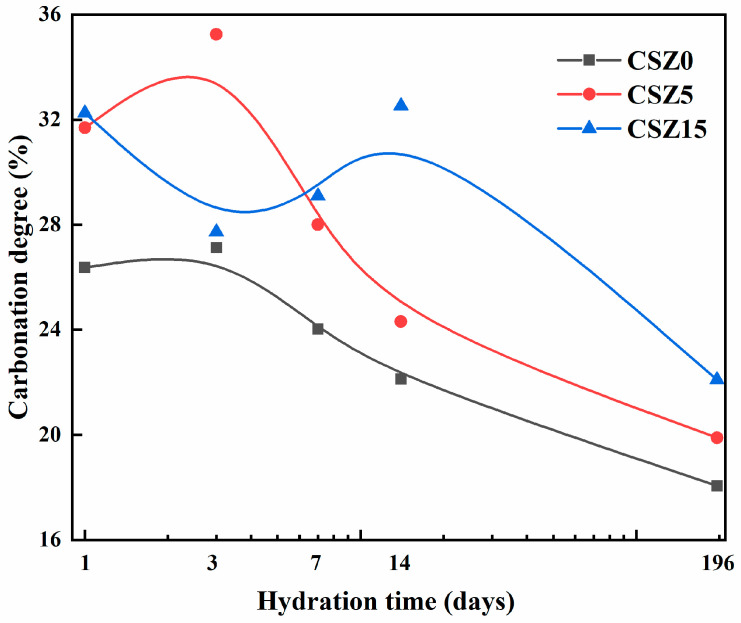
Carbonation degree of CSZ0, CSZ5, and CSZ15 specimens after different hydration time and then 2 h carbonation.

By changing the hydration curing time and zeolite substitution amount in the steel slag matrix, the CaCO_3_ amounts were fluctuations. To get further relationship of the two variables, the carbonation degrees of CSZ0, CSZ5, and CSZ15 specimens after different hydration times and then 2 h carbonation were presented in Figure 8. It was obvious to find that the carbonation degree of CSZ0 was lowest in the whole hydration time. Partial replacement of zeolite in steel slag was helpful for carbonation. Compare the carbonation degree of CSZ5 and CSZ15 specimens, in 1, 3, and 7 days hydration and subsequent 2 h carbonation, the optimum zeolite ratio in steel slag matrix was 5 wt.%. Continue to enhance the hydration time to 14 and 196 days, the optimum one turned to be 15 wt.%. The reason was explained in XRD analysis (see Section 3.2). Meanwhile, increase the hydration curing time and then carbonation would reduce the carbonation degree of SZ specimens, owing to the consumption of the excess mineral of β-C_2_S and Ca(OH)_2_. Three days hydration and then 2 h carbonation was the optimum curing method for SZ0 and SZ5 in carbonation degree. However, the strength values (see Figure 4) of CSZ0-3d and CSZ5-3d were lower than CSZ0-1d and CSZ5-1d, respectively. The carbonation degree and compressive strength were not linear relationships [28]. The reason would be explained at micro-structure analysis in detail (see Section 3.5).

### 3.4. Pozzolanic Reaction

**Figure 9:**
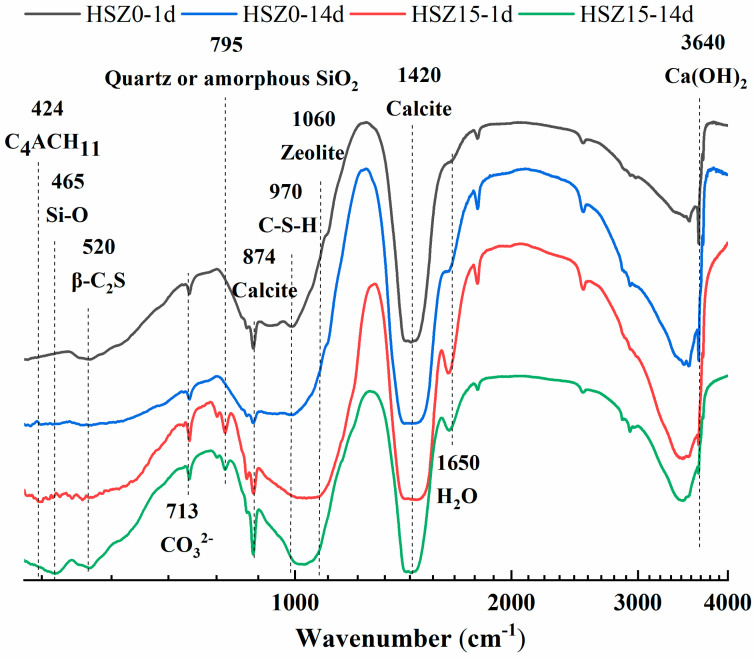
Fourier transform infrared spectroscopy (FT-IR) spectra of HSZ0 and HSZ15 after 1 day and 14 days of hydration.

FT-IR spectra were useful in acquiring information about the pozzolanic reaction and hydration products such as C–S–H. Figure 9 represented the FT-IR spectra of HSZ0 and HSZ15 after 1 day and 14 days hydration. In steel slag matrix, the CaCO_3_ minerals are expected to present a strong C–O vibration band in 731 cm^−1^ (calcite or aragonite) [35], 874 cm^−1^ (calcite) [36,37], and around 1420 cm^−1^ (calcite) [37,38]. And the Si–O asymmetric stretching vibration band around 520 and 975 cm^−1^ were related to β-C_2_S [37] and C–S–H [35]. And a weak stretching vibration band at 795 cm^−1^ could be assigned to quartz or amorphous SiO_2_ [39]. The O–H bending vibrations in the region 1600–3700 cm^−1^ were assigned to the presence of absorbing and crystal water, including OH^–^ in Ca(OH)_2_ at 3640 cm^−1^ [38]. And zeolite phases present a strong Si–O strength vibration around 1060 cm^−1^ [39] and the in-plane (460 cm^−1^) Si–O bending vibrations changes as results of polymerization of SiO_4_^4–^ unit in steel slag [29] and Si-gel [35]. Meanwhile, the bending vibrations in 424 cm^−1^ (AFmc) [30] was also detected.

After 14 days of hydration, the wavenumber at 1060 cm^−1^ (zeolite) in HSZ15-1d shifted to a lower one. And the O–H bending vibrations at 3640 cm^−1^ (Ca(OH)_2_) weakened dramatically. Meanwhile, the wavenumber at 970 cm^−1^ moved towards a higher wavenumber. These phenomena revealed the pozzolanic reaction in HSZ-14d specimens in Equations (7)–(9). The reduction of stretching vibration band intensities at 795 cm^−1^ (quartz or amorphous SiO_2_) could prove the pozzolanic reaction further in Equation (6). Compared to HSZ0-1d, the stretching vibration intensities at 1650 cm^−1^ enhanced in HSZ15-1d, owing to the existence of zeolitic water [39]. However, after 14 days hydration, it was clear to find the decline of stretching vibration intensities of zeolitic water, owing to participate in the hydration and pozzolanic reaction. The vibrations at 874 and 1420 cm^−1^ were assigned to the presence of calcite. The broad peak in 1420 cm^−1^ was due to the appearance of amorphous calcium carbonate (ACC, CaCO_3_·nH_2_O) [28,37,40]. HSZ0-1d and HSZ15-14d had sharper calcite peaks than HSZ0-14d and HSZ15-1d in 1420 cm^−1^. It revealed that increasing hydration time would increase the ACC content in HSZ0. However, the phenomena was opposite to HSZ15 due to produced AFmc minerals (see QXRD analysis in detail). So reasonable hydration time was essential for SZ specimens.

### 3.5. Microstructural Measurement

#### 3.5.1. MIP Analysis

**Figure 10:**
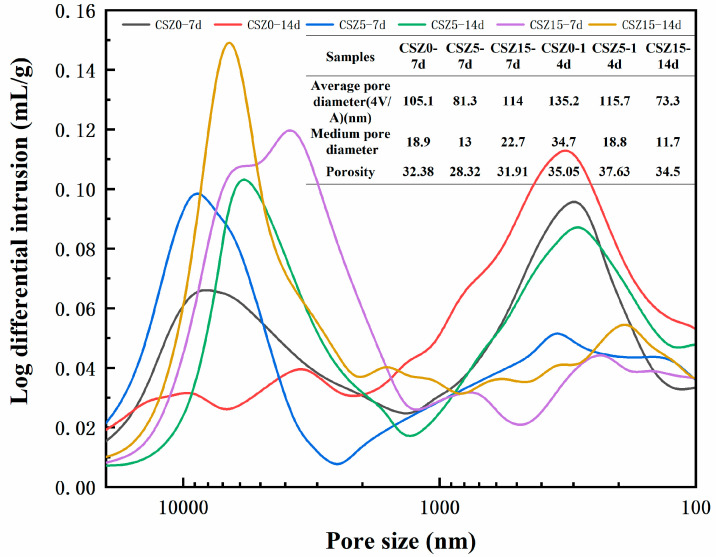
Pore size distribution of CSZ specimens after different hydration curing time and then 2 h carbonation.

To better understand the effect of compressive strength and pore size distribution, MIP results were presented in Figure 10. The medium pore diameters of CSZ0-7d, CSZ5-7d, CSZ15-7d, CSZ0-14d, CSZ5-14d, and CSZ15-14d were 18.9, 13, 22.7, 34.7, 18.8, and 11.7 nm, respectively. These data were consistent with compressive strength value, respectively (see Figure 4). Prolonging the hydration to 14 days, the porosity of SZ specimens increased compared with SZ-7d specimens to affect the strength. The pore above 5000 nm was formed during the molding and hydration curing process. The pore size peak at 3813 nm in CSZ15-7d was affirmed as zeolite pore by previous work results and the comparison of other CSZ0 and CSZ5 specimens. The peak was not obvious in CSZ5 specimens owing to the low content of zeolite. The pore size peak at 3813 nm in CSZ15-7d disappeared after prolonging the hydration time to 14 days, showing the crystalline zeolite participated in the pozzolanic reaction. The pore size in the range of 100–1000 nm was affected by hydration and carbonation curing. In this interval, the microporosities of CSZ0-7d and CSZ0-14d were higher than that of others. Meanwhile, CSZ0-14d performed the higher microporosity than that of CSZ0-7d. So, prolonging the hydration time would increase the microporosity to weaken the strength of steel slag specimens. On the contrary, the microporosities of CSZ5-7d, CSZ15-7d, and CSZ15-14d were lower than CSZ0-7d and CSZ0-14d even if the microporous volume of zeolite was 4.3 times than that of steel slag (see Figure 1), owing to the pozzolanic reaction and improving carbonation degree (see XRD and TGA analysis in detail). According to the MIP results, the porosity had a good relationship with compressive strength.

#### 3.5.2. FE-SEM Analysis

**Figure 11:**
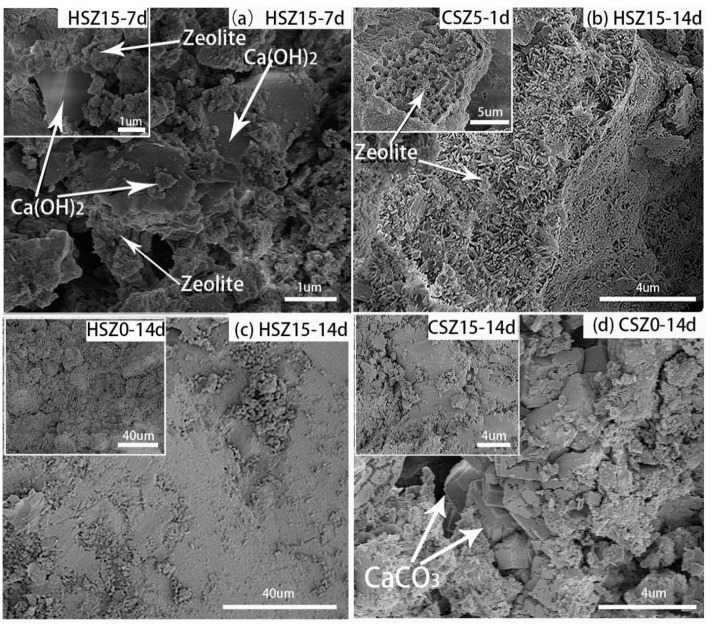
Field emission scanning electron microscopy (FE-SEM) morphologies of SZ specimens after the “H” or “C” process. (**a**) pozzolanic reaction of zeolite and Ca(OH)_2_ in HSZ15-7d; (**b**) morphology of zeolite after “H” and “C” process; (**c**) the morphology of surface density in HSZ0-14d and HSZ15-14d; and (**d**) morphology of CSZ0-14d and CSZ15-14d. (H: hydration at different times; C: hydration at different time and subsequent 2 h carbonation).

Figure 11 showed the FE-SEM images of SZ specimens after the “H” or “C” process. After 7 days hydration, the Ca(OH)_2_ reacted with zeolite partially in Equation (6) (pozzolanic reaction) as shown in Figure 11a. Prolonging the hydration time to 14 days, the crystalline zeolite participated in pozzolanic reaction in Equations (8) and (10) as shown in Figure 11b. Previous work had proved the porous bulk was zeolite by Energy Dispersive Spectrometer (EDS) analysis (CSZ5-1d). However, in HSZ15-14d specimens, the needle-like minerals in zeolite appeared, affirmed as AFmc by EDS analysis results (Ca, 20.17 wt.% and Al, 3.23 wt.%). Compared to CSZ5-1d, it was obvious to find that AFmc in HSZ15-14d was produced in the later hydration, owing to lower activity of crystalline zeolite. At the same time, AFmc was not observed in CSZ15-14d, owing to its participation in carbonation reaction in Equation (11). This phenomenon was consistent with QXRD analysis. Also, the pozzolanic reaction was beneficial to the surface density of SZ specimens as shown in Figure 11c. After 14 days hydration, the surface of HSZ15 was more smooth and denser than that of HSZ0. After 2 h carbonation curing, the surface of CSZ15-14d also presented more smoothly than that of CSZ0-14d as shown in Figure 11d even if CSZ0-14d produced much cube-like calcium carbonates for pore filling. It could slightly illustrate the compressive strength value of CSZ-14d specimens by Figure 11c,d.

## 4. Conclusions

Steel slag substituted by zeolite partially was beneficial for improving the compressive strength and carbonation degree of SZ specimens, owing to the pozzolanic reaction. Substitution ratios of 0, 5, and 15 wt.% were selected by previous work results. Before carbonation, how many days hydration for pozzolanic reaction was worthy to investigate. This study has successfully found the relationship with hydration time, strength, carbonation degree, and substitution ratio in SZ specimens. And the mechanism about pozzolanic reaction was studied further by XRD, TGA, FT-IR, and MIP methods. The following conclusions can be drawn.

The strength values of HSZ5 and HSZ15 were higher than that of HSZ0 in the late hydration process. Prolonging the hydration time would reduce the carbonation degree and compressive strength value of CSZ specimens. And the optimum curing process was 1 day hydration and then 2 h carbonation, considering the carbonation degree and strength of SZ specimens both.

The pozzolanic reaction could be divided into two-step. First, the active SiO_2_ reacted with Ca(OH)_2_ in the early hydration. Second, the skeleton structure of zeolite deteriorated in an alkaline environment and then participated in pozzolanic reaction in Equations (7)–(9). FT-IR results showed the reduction of stretching vibration band intensities at 795 cm^−1^ (quartz or amorphous SiO_2_) could prove the first step of pozzolanic reaction in SZ specimens in Equation (6). The MIP results presented that the zeolitic pore size peak at 3813 nm in CSZ15-7d disappeared after enhancing the hydration time to 14 days, showing the crystalline zeolite participated in the pozzolanic reaction.

A new mineral (AFmc) formed in HSZ15-14d in Equation (10) was observed in FE-SEM images. And also, the diffraction peak of AFmc was observed by XRD and QXRD analysis. Meanwhile, the strength value of CSZ15-14d was higher than that of CSZ0-14d and CSZ5-14d, owing to carbonated AFmc in Equation (11) by QXRD and TGA results.

## Figures and Tables

**Table 1:** Chemical composition of raw materials (wt.%).

Sample	CaO	SiO_2_	Fe_2_O_3_	Al_2_O_3_	MgO	MnO	TiO_2_	Total
Steel Slag	47.21	15.61	19.26	4.24	6.24	1.85	1.00	95.41
Zeolite	4.38	77.25	1.12	12.41	1.27	0.04	0.24	96.71

**Table 2:** Mineral content of raw materials (wt.%)**.**

Sample	Brownmillerite	Calcite	Larnite	Periclase	Portlandite	Wuestite	Lime	ACn
Steel Slag	12.1	8.5	15.6	9.2	5.6	3.7	1.3	44.0
-	**Clinoptilolite**		**Quartz**			**ACn**		
Zeolite	30.5		15.5			54		

**Table 3:** Components of the mix proportions of slag partially substituted by zeolite (SZ) specimens.

Curing Type	Abbreviated Names					Components (g)			Weight of Cubes (g) (2 cm × 2 cm × 2 cm)	Molding Pressure (MPa)
	Hydration Curing Periods					Steel Slag	Zeolite	Water		
	1 Day	3 Days	7 Days	14 Days	196 Days					
H ^a^	HSZ0-1d	HSZ0-3d	HSZ0-7d	HSZ0-14d	HSZ0-196d	20	0	2.6	20.0	8
	HSZ5-1d	HSZ5-3d	HSZ5-7d	HSZ5-14d	HSZ5-196d	19	1	2.6	19.3	8
	HSZ15-1d	HSZ15-3d	HSZ15-7d	HSZ15-14d	HSZ15-196d	17	3	2.6	17.6	8
H+C ^b^	CSZ0-1d	CSZ0-3d	CSZ0-7d	CSZ0-14d	CSZ0-196d	20	0	2.6	20.0	8
	CSZ5-1d	CSZ5-3d	CSZ5-7d	CSZ5-14d	CSZ5-196d	19	1	2.6	19.3	8
	CSZ15-1d	CSZ15-3d	CSZ15-7d	CSZ15-14d	CSZ15-196d	17	3	2.6	17.6	8

^a^ hydration curing; ^b^ 2 h carbonation curing.

**Table 4:** Phases and crystallography open database (COD) codes used for Rietveld analysis.

Phase	Formula	Abbr.	COD Codes
Larnite/Belite	Ca_2_SiO_4_	β-C_2_S	9012792
Calcite	CaCO_3_	CČ	9016706
Clinoptilolite	(K,Na,Ca,Mg)_2.52_[(AlO_2_)_3.08_ (SiO_2_)_14.92_]·15H_2_O	-	9008265
Portlandite	Ca(OH)_2_	CH	9009098
Periclase	free-MgO	f-MgO	9008671
Zincite	ZnO	-	9008877
Aragonite	CaCO_3_	CČ	2100187
Vaterite	CaCO_3_	CČ	9016215
Lime	free-CaO	f-CaO	1011327
Quartz	SiO_2_	-	1011172
Wuestite	Fe_0.929_O	-	1011165

The cut slices were prepared from the specimens, dried in a vacuum chamber at 50 °C for 24 h, and platinized to imaging.
